# Macular Xanthophylls Are Related to Intellectual Ability among Adults with Overweight and Obesity

**DOI:** 10.3390/nu10040396

**Published:** 2018-03-23

**Authors:** Naiman A. Khan, Anne. M. Walk, Caitlyn G. Edwards, Alicia R. Jones, Corinne N. Cannavale, Sharon V. Thompson, Ginger E. Reeser, Hannah D. Holscher

**Affiliations:** 1 Department of Kinesiology and Community Health, University of Illinois, Urbana, IL 61801, USA; amcclur3@illinois.edu (A.M.W.); covello@illinois.edu (A.R.J.); reeser2@illinois.edu (G.E.R.); hholsche@illinois.edu (H.D.H.); 2 Division of Nutritional Sciences, University of Illinois, Urbana, IL 61801, USA; cgedwar2@illinois.edu (C.G.E.); svthomp2@illinois.edu (S.V.T.); 3 Neuroscience Program, University of Illinois, Urbana, IL 61801, USA; cannava2@illinois.edu; 4 Department of Food Science and Human Nutrition, University of Illinois, Urbana, IL 61801, USA

**Keywords:** adiposity, retinal, carotenoids, cognitive function, lutein, zeaxanthin, carotenoids

## Abstract

Excess adiposity or obesity has been inversely related to cognitive function and macular xanthophyll status. However, whether the neuroprotective effects of macular xanthophylls on cognitive function are independent of excess adiposity is unclear. We investigated the relationship between macular xanthophylls and intellectual ability among adults (*N* = 114) between 25 and 45 years with overweight and obesity (≥25 kg/m^2^). Dual energy X-ray absorptiometry and heterochromatic flicker photometry were used to assess whole body adiposity (%Fat) and macular pigment optical density (MPOD), respectively. Dietary xanthophylls (lutein and zeaxanthin) were assessed using 7-day diet records. The Kaufman Brief Intelligence Test-2 (KBIT-2) was used to assess general intelligence (IQ) as well as fluid and crystallized intelligence. Bivariate correlations revealed that MPOD was inversely related to %Fat and positively associated with IQ and fluid intelligence. Although %Fat was inversely correlated to IQ and fluid intelligence, this relationship did not persist following adjustment for sex and MPOD. Further, MPOD was an independent predictor of IQ and fluid intelligence. However, no significant relationships were observed between MPOD and crystalized intelligence. These results suggest that macular xanthophylls are selectively related to fluid intelligence, regardless of degree of adiposity among adults with overweight and obesity.

## 1. Introduction

The prevalence of obesity has increased three-fold since the 1980s and currently affects approximately 40% of the US population [1]. Excess fat mass or adiposity is known to directly contribute to a wide range of metabolic disorders and chronic diseases including type 2 diabetes and cardiovascular disease, as well as certain cancers [2]. Overweight and obesity are also related to mood disorders including anxiety and depression [3]. Increasing evidence suggests that the consequences of obesity extend beyond physical and psychological health into cognitive function and brain health [4]. For example, overweight and obesity are associated with dementia in older age [4,5]. Although the mechanistic underpinnings of the adiposity–cognition relationship remain inadequately characterized, it is likely that excess adiposity influences cognitive function via both direct and indirect pathways. Suboptimal nutritional intake and status have received considerable interest as a potential behavioral mechanism by which adiposity may detrimentally influence cognitive function. Elevated weight status is often, though not always, accompanied by poorer self-reported nutritional intake and blood biomarkers indicative of lower consumption of nutrient-rich foods such as fruits and vegetables [6]. Diet is thought to influence cognitive function by a variety of potential mechanisms that include, but are not limited to, providing essential nutrients for brain development [7], amelioration of neuroinflammation [8,9], and provision of energy [10]. However, although epidemiological and cognitive neuroscience literature indicates that nutritional status plays a role in cognitive function and brain health, the specificity of the relationships between particular dietary components and their implications for select aspects of cognitive function are unclear.

Given that the human retina and other neural tissues in the brain disproportionately accumulate xanthophylls, particularly lutein, the study of xanthophylls and their cognitive implications has gained increasing importance [11]. Within the eye, lutein along with two other xanthophylls, zeaxanthin and meso-zeaxanthin, forms macula pigmentation that protects retinal tissue from photooxidative damage [12]. Opportunely, macular pigment optical density (MPOD)—a noninvasive measure of retinal and brain lutein [13]—can be utilized as a biomarker for brain health. Although MPOD reflects the cumulative concentration of carotenoids in the retina, lutein is the source of two of the three xanthophylls [14]. However, although lutein and zeaxanthin are often found in many of the same foods (e.g., dark green leafy vegetables), lutein comprises over five-fold greater proportion in diet, relative to zeaxanthin [15]. Pertinent to the work presented here, xanthophylls are fat soluble and sequestered in adipose tissue. Indeed, previous work has shown that individuals with elevated BMI (≥29 kg/m^2^) exhibit over 20% less MPOD [16] and that the degree of adiposity is inversely associated with MPOD [16,17]. However, although, higher MPOD has been associated with greater cognitive function across multiple domains [18,19,20], the relationship between macular xanthophylls and intellectual ability among adults with overweight and obesity has not been directly examined.

Intelligence represents a critical cognitive ability that is known to support higher-order cognitive processes such as executive function as well as the acquisition of knowledge and learning across the lifespan [21]. Intelligence can be studied as general intelligence (i.e., intelligence quotient (IQ)) as well as its related but dissociable components, including fluid and crystallized intelligence. Fluid intelligence encompasses the ability to conduct adoptive problem solving in novel situations, and represents the capacity to creatively and flexibly contend with everyday challenges without relying on prior knowledge [22]. On the other hand, crystallized intelligence refers to the ability to retrieve and use information that has been acquired throughout life [23]. It is important to study specific constructs of intelligence given that fluid and crystallized intelligence exhibit differential susceptibility to factors such as aging. For example, aging has a profound effect on decline in fluid intelligence over the lifespan whereas crystallized intelligence appears to be spared, and may even improve with age [24,25]. In the context of nutrition, studying the influence of macular xanthophylls in relation to different constructs of intelligence may provide insights into the value of lifetime dietary consumption of lutein and zeaxanthin rather than the acute effects of nutrients on preventing age-related decline [24,26]. Although previous work has indicated that serum lutein is related to crystallized intelligence among older adults [26], the relationship between macular xanthophyll status and crystallized and fluid intelligence has thus far remained unexamined. 

Previous research has revealed differential relationships between xanthophyll carotenoids and adiposity and cognitive abilities; however, it is not clear whether the relationship between MPOD and cognition is independent of adiposity. Accordingly, the present work aimed to investigate the relationship between MPOD and different measures of intellectual abilities (IQ, fluid and crystallized intelligence) among adults with overweight and obesity. Given prior work indicating a positive influence of MPOD on other domains of cognitive function—known to be related to intellectual abilities—we hypothesized that greater macular xanthophyll status would be positively associated with all measures of intelligence. Additionally, we anticipated that these relationships would persist even after accounting for demographic factors, adiposity, and dietary consumption of lutein and zeaxanthin. 

## 2. Materials and Methods

### 2.1. Participants

One hundred and fourteen participants were recruited from the East-Central region of Illinois through the use of flyers posted in public buildings. All subjects provided informed consent for inclusion before they participated in the study. The study was conducted in accordance with the Declaration of Helsinki, and the protocol was approved by the Ethics Committee of the University of Illinois (Project identification codes 16071 and 16277). Participants were excluded if they were pregnant, had a history of neurological disease, used anti-psychotic or anti-anxiety medication, had a history of chronic metabolic diseases, or had non-normal or uncorrected vision based on the minimal 20/20 standard

### 2.2. Measures

#### 2.2.1. Anthropometrics and Adiposity Assessment

Body mass index (BMI (kg/m^2^)) was assessed using standing height and weight measurements with participants wearing lightweight clothing and no shoes. A stadiometer (model 240; SECA, Hamburg, Germany) and a digital scale (WB-300 Plus; Tanita, Tokyo, Japan) were used to measure height and weight, respectively. The average of three measurements were used for final analyses. Adipose tissue was measured by dual energy X-ray absorptiometry (DXA) using a Hologic Horizon W bone densitometer (APEX Software version 5.6.0.5; Hologic, Bedford, MA). Whole body percent body fat (%Fat) was assessed using the standard Hologic software, as previously described [27].

#### 2.2.2. Dietary Lutein and Zeaxanthin Assessment 

Participants were asked to record all beverages and foods consumed for at least 7 days using a diet record/food log to assess habitual dietary consumption of xanthophyll carotenoids (i.e., lutein and zeaxanthin). Participants were provided with a food log with detailed instructions given by a trained member of the research staff at the completion of their first laboratory visit. The diet records were returned at a subsequent laboratory visit. In addition, the record contained written instructions for recording food intake (including how to describe food preparation methods, added fats, brand names, and ingredients of mixed dishes and recipes). Trained staff entered food records into the Nutrition Data Systems-Research Version 2015 (Nutrition Coordinating Center (NCC), University of Minnesota, Minneapolis, MN, USA) software. Current dietary databases lack the appropriate information to ascertain valid dietary intakes of the individual xanthophylls, therefore, an aggregate measure of both lutein and zeaxanthin was used. Nutrient-level analyses were conducted by using the intake properties file to determine average daily consumption of lutein and zeaxanthin. 

#### 2.2.3. Macular Pigment Optical Density

MPOD was assessed via a customized hetero-flicker photometry (cHFP) technique administered using a macular densitometer (Macular Metrics Corporation, Rehoboth, MA, USA). The principles of this technique have been previously described [28]. Briefly, participants were asked to view stimuli peaking at a measuring wavelength of 460 nm that flickers in counterphase with a 570 nm reference (flicker rate being optimized for the optimal width of the subject’s null zone). Participants were asked to adjust the radiance to identify a null flicker zone by indicating when they could no longer detect the flicker. The task is done while the stimulus is centrally fixated (measuring macular pigmentation where it is most dense) and at 7 degrees in the para-fovea (where density is minimal). The MPOD is calculated by subtracting the foveal from the parafoveal log sensitivity measurements after normalizing at 570 nm.

#### 2.2.4. Intellectual Ability Assessment 

The Kaufman Brief Intelligence Test, Second Edition (KBIT-2) was used to assess intelligence quotient (IQ), as well as fluid and crystallized intelligence. The KBIT-2 has been nationally normed for ages 4–90 to assess general intellectual abilities and has been shown to have comparable scores to other intelligence scales [29,30]. The test is divided into three subtests and takes approximately 25–30 min to complete. The verbal knowledge subtest includes 60 questions where the participant responds by choosing which image is most associated with the word or question spoken by the researcher. The riddle subtest consists of 48 riddles that have a single word response. The matrices subtest has 46 logic problems where the participant must choose which of six pictures is most associated with a single stimulus picture or which picture best completes a 2 × 2, 2 × 3, or 3 × 3 matrix. In each of the subtests, correct answers are given a score of 1, and the total scores are converted into standard scores. In addition to a composite IQ score, the KBIT-2 yields a crystallized intelligence measure which includes the verbal knowledge and riddle subtests in addition to a fluid intelligence score, which is assessed with the matrices subtest. 

### 2.3. Statistical Analyses

All analyses were conducted using SPSS version 24 (IBM, Armonk, NY, USA). Any variables that were non-normally distributed were log transformed. Pearson’s *r* bivariate correlations were used to determine initial associations between demographic factors (age, sex, and income), dietary lutein and zeaxanthin, adiposity (%Fat), and intelligence outcomes (IQ, fluid, and crystallized). Subsequently, linear regression models were developed to explain variability in intelligence outcomes. All potential confounding variables (i.e., age, sex, income, dietary lutein and zeaxanthin, and %Fat) and MPOD were included in the same model to determine the relationship between MPOD and intelligence outcomes (i.e., dependent variables) following the adjustment of potentially confounding variables. Two-tailed tests are reported, with alpha of 0.05 for determining statistical significance. *R*^2^ is reported to indicate model fit, and standardized betas are reported to indicate the weight of respective variables within the model.

## 3. Results

### 3.1. Participant Characteristics and Descriptive Information 

Participant descriptive characteristics are summarized in Table 1. Participants ranged in age from 25 to 45 years (Mean 34.6 ± 6.1 years) with a greater proportion of females (61%) relative to males (39%). The majority of the participants (77%) were of moderate to higher socioeconomic status based on annual household income (>$41,000). Approximately, 80% of the participants were White or Caucasian while only 9% were Asian and 7% were Black or African American. A little over half of the participants (54%) had obesity based on BMI ≥ 30 kg/m^2^. There was considerable variability in self-reported dietary lutein and zexanthin intake (2319.7 ± 3503.0 µg); however, approximately 68% of the participants reported consuming less than 2000 µg/day. Nevertheless, the average consumption of lutein and zexanthin was comparatively greater in the current sample relative to consumption patterns assessed in a nationally representative sample (i.e., 791–1015 µg/day) [14]. Descriptive analyses of the overall IQ measure indicated that only one individual scored below 1 SD of the normed value (i.e., <85) whereas 40 participants (35%) scored over 1 SD of the normed values (i.e., >115). 

### 3.2. Bivariate Correlations

**Figure 1:**
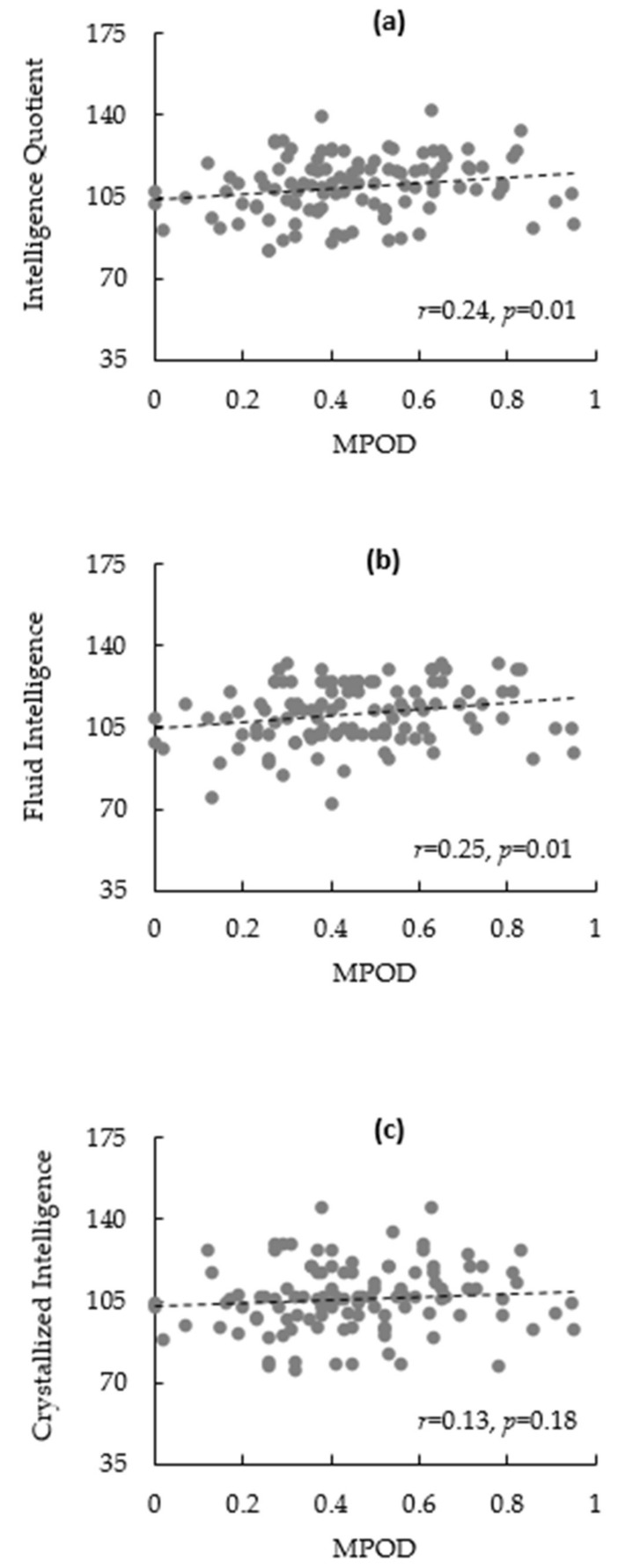
(**a**–**c**): Scatterplots illustrating relationships between macular pigment optical density (MPOD) and IQ (**a**); fluid intelligence (**b**); and crystallized intelligence (**c**).

The bivariate correlations are summarized in Table 2. There was a significant association between sex and %Fat (*r* = −0.77, *p* < 0.01), IQ (*r* = 0.26, *p* = 0.01), fluid intelligence (*r* = 0.23, *p* = 0.01), and crystallized intelligence (*r* = 0.20, *p* = 0.03). %Fat was inversely related to MPOD (*r* = −0.19, *p* = 0.04), IQ (*r* = −0.22, *p* = 0.02), and fluid (r = −0.24, *p* = 0.01), but not to crystallized intelligence (*r* = −0.13, *p* = 0.16). Scatterplots illustrating the correlations between MPOD and intelligence outcomes can be found in Figure 1a–c. However, dietary lutein and zeaxanthin was not significantly associated with any other variable. 

### 3.3. Regression Models

Regression analyses are summarized in Table 3. The regression model explaining variability in overall IQ (model *p* < 0.01) revealed that only MPOD (β = 0.20, *p* = 0.04) was an independent positive predictor of IQ. According to the model explaining variability in fluid intelligence (model *p* = 0.02), only MPOD (β = 0.20, *p* = 0.03) was a significant predictor. Finally, although the model explaining variability in crystallized intelligence approached statistical significance (*p* = 0.05), there were no significant individual predictors. 

## 4. Discussion

The present work aimed to delineate the relationship between macular xanthophylls (i.e., MPOD) and different measures of intellectual abilities among adults with overweight and obesity. Consistent with previous work, we observed that individuals with greater adiposity appeared to have lower MPOD [16]. However, we extend the knowledge in this area by demonstrating that MPOD was related to general IQ as well as fluid intelligence even after adjusting for sex and adiposity. Additionally, MPOD was selectively related to fluid intelligence since no significant relationships were observed for crystallized intelligence. Interestingly, while we initially observed a negative relationship between adiposity and intelligence outcomes in the bivariate correlations, these relationships were mitigated following inclusion of demographic factors and xanthophyll measures in the regression models. Therefore, an implication of the present work is that macular xanthophylls can serve as predictors of intellectual abilities among adults, irrespective of their degree of adiposity. 

The precipitous rise in prevalence of overweight and obesity since the latter half of the 20th century has emerged as a major public health concern due to its implications for both physical and mental health [31,32]. In addition to its metabolic consequences, excess adiposity has been linked to decrements in brain health and cognitive function, along with a higher risk for cognitive decline in older age [33,34]. Although the underlying mechanisms are unclear, evidence from neuroimaging studies indicates that excess adiposity is associated with alterations in brain structure and function that typically accompany cognitive deficits including reduced synaptic plasticity [35], reduced processing speed [36], and lower grey matter volume [37]. However, although a considerable body of literature has examined the influence of obesity on brain structure as well as multiple domains of cognitive function [32,38], few studies have directly examined the impact of excess adiposity on different measures of intelligence. Understanding the implications of excess adiposity for intellectual abilities is important because intelligence supports higher-order cognitive processes such as executive function as well as the acquisition of knowledge and learning across the lifespan [21]. 

Regarding the previous work on obesity and intelligence, a systematic review and meta-analysis conducted in 2010 revealed that only three studies had directly examined the relationship between obesity and any measure of intelligence among adults [39]. Although a negative influence of obesity on IQ was indicated, this relationship appeared to be mitigated following adjustment of demographic factors [40]. This finding is consistent with the observation in the current study since we also observed an inverse relationship between %Fat and all measures of intelligence, though, this relationship was no longer evident once we controlled for sex and MPOD. Given the paucity of data in this area, additional studies are needed to confirm these observations. Indeed, in a recent study among clinically healthy adults with varying weight status, Spyridaki and colleagues [41] observed an inverse association between elevated BMI and fluid intelligence, with obese participants displaying significantly poorer performance compared with age-matched normal-weight peers. Interestingly, this relationship was mediated by chronic low-grade inflammation as indicated by high-sensitivity C-reactive protein, erythrocyte sedimentation rate, and fibrinogen, providing potential insights into the inflammatory underpinnings of adiposity effects on fluid intelligence. Therefore, the influence of adiposity on intellectual abilities is perhaps driven, to a greater extent, by individual variability in nutritional status and metabolic risks associated with excess adiposity. 

Converging evidence from epidemiological and cognitive neuroscience suggests that nutritional intake and status may play important roles in neuroprotection and cognitive function across the lifespan [42]. However, the vast majority of Americans routinely fail to meet their recommended servings of dark green leafy vegetables [43] contributing to low intakes of the xanthophylls lutein and zeaxanthin [14]. Opportunely, unlike many dietary components, the amount of macular xanthophyll concentration can be measured directly or non-invasively as MPOD [44]. Pertinent to the work presented here, individuals with greater adiposity have been shown to have lower macular accumulation of xanthophylls [16,17]. Indeed, although carotenoids are found in many peripheral tissues in the human body—including the liver, ovary, testes, pancreas, kidney, and several tissues of the eye [45,46]—it has been estimated that more than 80% of the total carotenoids in the body are accumulated in adipose tissue. Therefore, adipose tissue may serve as a potential storage site for later xanthophyll usage [47] and it is possible that individuals with greater adipose tissue may sequester comparatively greater amounts of xanthophylls in peripheral adipose tissues, thereby limiting the availability for accumulation of xanthophylls by other tissues (e.g., retina) [16]. Consistent with this expectation, Hammond and colleagues [16] assessed BMI and %Fat (using bioelectric impedance) and observed small (*r* = −0.12) but inverse relationships with MPOD. However, this inverse relationship was driven by individuals with higher degree of adiposity (BMI > 29 kg/m^2^). Subsequent work by Nolan and colleagues demonstrated inverse relationships between %Fat, assessed using DXA, and MPOD among adult males [17]. However, the small size of the retina relative to total body adiposity, irrespective of obesity status, limits the plausibility of the hypothesis that whole body adiposity directly competes with retina for xanthophylls. Therefore, while the results of the present study are consistent with previous work indicating an inverse relationship between adiposity and MPOD among adults with overweight and obesity, additional supplemental and longitudinal studies examining changes in xanthophyll accumulation in adipose tissue, retina, as well as serum are needed to characterize the interaction between retinal xanthophylls and adiposity.

Although many studies in recent years have linked greater MPOD with multiple aspects of cognitive function [18], to our knowledge, this is the first study to examine the influence of both adiposity and macular xanthophylls and their implications for cognitive abilities among individuals with overweight and obesity. Further, the extent to which macular xanthophylls contribute to the intellectual abilities has not been directly investigated. Superior MPOD status in the current sample was related to higher general intelligence as indicated by IQ. This relationship was sustained even after adjusting for adiposity. General intelligence is traditionally conceptualized as the ability of one to solve complex problems or reason adaptively, abilities which influence many facets of life including decision-making abilities, vocational success [48], and social mobility [49]. Thus, the observed positive influence of MPOD on general intelligence potentially points to the importance of maintaining adequate macular xanthophyll status for a wide range of benefits for mental health and quality of life. 

Further, we observed a selective impact of MPOD for fluid, rather than crystallized, intelligence. Although previous studies have linked macular xanthophyll status to executive function processes–such as memory and complex attention—known to be associated with fluid intelligence [18,19,50], the data presented here are the first to directly link fluid intelligence to MPOD. Fluid intelligence encompasses the ability to conduct adaptive problem solving in novel situations, and represents the capacity to creatively and flexibly contend with everyday challenges without relying on prior knowledge [22]. The observed relationship between MPOD and fluid intelligence has important public health implications particularly in the context of cognitive aging. In the absence of neurodegenerative disease, age-related decline in fluid intelligence presents a considerable challenge for an increasingly aged population in western societies [51]. Relevant to the findings presented here, recent work indicates that nervous system health contributes to the variability in age-related decline in fluid intelligence [51]. Given that the brain is predominantly composed of long-chain polyunsaturated lipids, with a highly oxygenated structure predisposed to inflammatory stress, nutrition has the potential to either provide neuroprotection against or precipitate the oxidative and inflammatory processes in the brain [52]. For example, dietary consumption of long-chain omega-3 fatty acids has been shown to improve white matter microstructural integrity and gray matter volume in frontal and parietal cortices [53], brain regions known to support fluid intelligence [54,55]. Lutein, being a potent antioxidant with inflammatory properties, may have overlapping neuroprotective effects for general and selective domains of cognitive function. For example, lutein has been found to selectively co-localize in brain membranes with omega-3 fatty acids; therefore, it is possible that the implications of lutein for fluid intelligence are mediated by sparing vital omega-3 fatty acids such as docosahexaenoic acid (DHA) from oxidation, and preserving membrane fluidity [55]. Additional plausible mechanisms of lutein action may include its role in maintaining structural integrity and function of brain membranes and axonal projections associated with neural substrates known to be vital for memory and learning (e.g., hippocampus and prefrontal cortex), as well as gene regulation involved in myelin synthesis [56,57]. 

Contrary to our a priori hypothesis, MPOD was not found to be predictive of crystallized intelligence above and beyond demographic factors. Crystallized intelligence relies on using previously learned verbal knowledge and is amenable to educational status and prior learning history. While the reason for the specificity of MPOD to fluid intelligence in our study is unclear, it may be related to developmental timing or to the brain regions underlying the task demands of crystallized compared to fluid intelligence. Regarding developmental timing, the majority of studies that have examined cognition and lutein status have been conducted in older adults, a population which may already be experiencing cognitive decline. Crystallized intelligence has been shown to be less amenable to cognitive aging; in fact, a seminal study by Horn and Cattell (1967) demonstrated that fluid intelligence is likely to decline with age, whereas crystallized intelligence may increase [22]. Renzi et al. (2014) showed that in adult participants experiencing mild cognitive impairment, MPOD was related to language function (a reasonable proxy of crystallized intelligence), but in healthy older adults MPOD was related only to visuo-spatial and construction abilities, which are more on par with fluid intelligence [58]. Thus, we may be less likely to see a relationship between MPOD and crystallized intelligence in participants who are cognitively healthy, young to middle-aged adults, as seen in our sample. Regarding neural correlates, the evidence is much more speculative. Work has shown that structural correlates unique to fluid intelligence include more voxels in the frontal lobe, whereas correlates unique to crystallized intelligence include more voxels in the occipital lobe [59]. However, despite the unique architecture, there was considerable overlap of brain structure between the two, as well as overlap of fluid and crystallized intelligence with the structures involved in general intelligence. While lutein has been found in both frontal and occipital regions [11,60], it is currently unknown whether its effects are exerted equally across the brain regions in which it is found. Therefore, it is possible that lutein may affect brain areas differentially, scaffolding cognitive function associated with certain brain regions preferentially over others. However, considerably more work is needed to unravel the complex relationships between the presence of lutein in the brain, structural and functional changes exerted by lutein in the brain, and corresponding changes to cognitive function. 

Findings from recent neuroimaging studies provide support for the neuroprotective role of xanthophylls for brain function. In a recent study by Hammond and colleagues [61], MPOD was related to greater white matter integrity in the uncinate fasciculus and cingulum. Further, serum lutein and zeaxanthin status was associated with white matter tracts vulnerable to age-related decline. Additionally, lutein supplementation appears to moderate cognitive decline in verbal learning and may benefit neurocognitive function by enhancing cerebral perfusion among older adults [62]. Thus, the preferential accumulation of lutein across brain cortices and membranes indicates that, relative to other carotenoids, lutein is uniquely suited to support fluid intelligence through a variety of mechanisms. One of the novel implications of the present work is that it demonstrates that the positive implications of macular xanthophylls for intellectual abilities are evident even among adults with overweight and obesity or individuals with greater risk for suboptimal macular xanthophyll status. 

Counter to our a priori expectations, there was no association between dietary xanthophylls and MPOD. In a previous study among a large sample of mid-western adults (*N* = 278), Curran-Celentano and colleagues [63] observed a positive association between dietary lutein and zeaxanthin, assessed using food frequency questionnaires, and MPOD. However, this relationship was not as robust as anticipated (*r* = 0.25) and the differential findings of the two studies may be attributed, at least in part, to differences in methodology (i.e., food records vs. food frequency questionnaires) as well as sample characteristics in MPOD and dietary xanthophyll intake. For example, the MPOD values observed in the current study were over two-fold greater (0.46 ± 0.21 vs. 0.21 ± 0.13) than the aforementioned study. This difference extended to the reported lutein and zeaxanthin intake as well since the intake reported in the present study was also over two-fold greater compared to the previous study (2261 ± 3437 µg vs. 1101 ± 838 µg). Therefore, it is possible that our study was inadequately powered to observe this relationship. Additionally, in another study, Nolan and colleagues also observed a positive relationship between only dietary zeaxanthin and MPOD by using food frequency questionnaires [17]. Given that the current work relied on diet records, it is possible that food frequency questionnaires may be more suitable for studying the relationship between dietary and macular xanthophylls. Further, it is plausible that we were not able to detect a relationship since we did not assess pigmentation across the entire spatial profile of the macular. It is possible that self-reported dietary xanthophylls could have differential relationships with pigmentation at different spatial locations of the retina. Errors in measurement may have also contributed to these null findings given the limitations and problems associated with the use of self-report diet measures and their validity [64,65]. Indeed, errors in misreporting may be particularly relevant to the current work since previous work indicates that errors in misreporting on dietary self-reports are often more pronounced among adults with overweight and obesity [66]. Finally, future work that includes direct or indirect assessment of energy expenditure is necessary to account for the plausibility of the self-reported intakes.

## 5. Conclusions

Although a considerable body of literature has linked superior macular xanthophyll status to multiple aspects of cognitive function, previous work has not directly examined the influence of MPOD on constructs of intellectual ability. The results of the present work reveal both selective and general relationships between MPOD and intellectual abilities. Further, we demonstrate these relationships among individuals with overweight and obesity, known to be at risk for lower MPOD status. Given that excess fat mass has also been related to poorer cognitive function and brain health, the finding that MPOD was positively related to intelligence provides a potential opportunity to counter obesity-related cognitive impairment using dietary approaches. 

## Figures and Tables

**Table 1:** Participant characteristics.

Variable	Mean ± SD
Age, years	34.3 ± 6.1
Sex	69 (F), 45 (M)
Income	
Low [<$41,000], *n* (%)	37 (32)
Medium [$41,000*–*$70,000], *n* (%)	36 (32)
High [>$70,000], *n* (%)	41 (36)
Dietary Lutein and Zeaxanthin, µg	2319.7 ± 3503.0
Body Mass Index, kg/m^2^	31.9 ± 5.3
Overweight, *n* (%)	55 (48)
Obese, *n* (%)	59 (52)
Whole body Adiposity, %	37.7 ± 8.9
Macular Pigment Optical Density	0.46 ± 0.21
Intelligence Quotient	109.5 ± 12.9
Crystallized Intelligence	110.8 ± 12.9
Fluid Intelligence	106.0 ± 15.0

**Table 2:** Bivariate correlations between demographics, diet, adiposity, MPOD, and intelligence.

		1	2	3	4	5	6	7	8
1. Age	*r*								
	*p*								
2. ^a^ Sex	*r*	−0.04							
	*p*	0.68							
3. Income	*r*	0.50 **	−0.05						
	*p*	0.00	0.57						
4. Dietary LZ	*r*	−0.08	−0.01	0.09					
	*p*	0.38	0.88	0.33					
5. %Fat	*r*	0.06	−0.77 **	0.06	−0.09				
	*p*	0.55	0.00	0.52	0.35				
6. MPOD	*r*	−0.18	0.18	−0.06	0.06	−0.19 *			
	*p*	0.06	0.06	0.55	0.52	0.04			
7. IQ	*r*	0.02	0.26 **	0.15	0.16	−0.22 *	0.24 *		
	*p*	0.81	0.01	0.12	0.08	0.02	0.01		
8. Fluid Intelligence	*r*	−0.01	0.23 *	0.08	0.14	−0.24 *	0.25 **	0.80 **	
	*p*	0.95	0.01	0.38	0.14	0.01	0.01	0.00	
9. Crystallized Intelligence	*r*	0.11	0.20 *	0.18	0.13	−0.13	0.13	0.85 **	0.36 **
	*p*	0.27	0.03	0.05	0.16	0.16	0.18	0.00	0.00

MPOD, macular pigment optical density; %Fat, whole body adiposity; LZ, dietary lutein and zeaxanthin; IQ, intelligence quotient. * Significant at *p* ≤ 0.05 (two-tailed); ** Significant at *p* ≤ 0.01 (two-tailed); ^a^ Females coded as 0 and males coded as 1.

**Table 3:** Regression analyses explaining variability in intelligence outcomes.

	Intelligence Quotient			Fluid Intelligence			Crystallized Intelligence		
	β	*p*	*Model R*^2^	β	*p*	*Model R*^2^	β	*p*	*Model R*^2^
			0.15 **			0.13 *			0.11
Age	<0.01	1.00		<0.01	0.99		0.06	0.56	
Sex	0.24	0.09		0.13	0.37		0.27	0.07	
Income	0.16	0.14		0.10	0.37		0.15	0.16	
Dietary LZ	0.14	0.12		0.11	0.23		0.13	0.17	
%Fat	0.01	0.93		−0.09	0.52		0.09	0.53	
MPOD	0.20 *	0.04		0.20 *	0.03		0.11	0.25	

* Significant at *p* ≤ 0.05; ** Significant at *p* ≤ 0.01.
